# Enhanced amygdala-frontal operculum functional connectivity during rest in women with chronic neck pain: Associations with impaired conditioned pain modulation

**DOI:** 10.1016/j.nicl.2021.102638

**Published:** 2021-03-22

**Authors:** Iris Coppieters, Barbara Cagnie, Robby v, Mira Meeus, Inge Timmers

**Affiliations:** a Pain in Motion Research Group VUB (PAIN), Department of Physiotherapy, Human Physiology and Anatomy, Faculty of Physical Education and Physiotherapy, Vrije Universiteit Brussel, Brussels, Belgium; b Department of Physical Medicine and Physiotherapy, University Hospital Brussels, Brussels, Belgium; c Pain in Motion International Research Group, Belgium; d Department of Rehabilitation Sciences, Faculty of Medicine and Health Sciences, Ghent University, Belgium; e Department of Rehabilitation Sciences and Physiotherapy, Faculty of Medicine and Health Sciences, University of Antwerp, Belgium; f Department of Rehabilitation Medicine, Maastricht University, Maastricht, Netherlands; g Department of Anesthesiology, Perioperative, and Pain Medicine, Stanford University, Stanford, CA, United States

**Keywords:** Resting-state functional connectivity, Pain processing, Chronic whiplash associated disorders, Chronic idiopathic neck pain, Quantitative sensory testing

## Abstract

**Background:**

Chronic neck pain is a leading cause of disability worldwide, affecting the lives of millions of people. Research investigating functional brain alterations in relation to somatosensory function is necessary to better understand mechanisms underlying pain development and maintenance in individuals with chronic neck pain, yet remains scarce. This case-control study aimed to examine resting-state functional connectivity alterations and associations with pain outcomes, self-reported central sensitization-related symptoms and quantitative sensory testing (QST) measures in patients with chronic non-traumatic (idiopathic/CINP) neck pain and chronic traumatic (whiplash associated/CWAD) neck pain compared to pain-free controls.

**Methods:**

Resting-state functional magnetic resonance images were acquired in 107 female participants (38 CINP, 37 CWAD, 32 healthy controls). After data pre-processing, seed-to-seed analyses were conducted focusing on resting-state functional connectivity involving pre-defined regions of interest that have previously been observed to be structurally or functionally altered and/or associated with pain-related measures in this patient population.

**Results:**

Findings demonstrate enhanced left amygdala functional coupling during rest with the left frontal operculum in women with CINP and CWAD compared to controls. This increased resting-state functional connectivity was associated with more self-reported symptoms related to central sensitization and decreased efficacy of conditioned pain modulation. Furthermore, enhanced connectivity between the left amygdala and left frontal orbital cortex, and between the left pallidum and the left frontal operculum was observed only in patients with CWAD compared to healthy controls. In patients, additional associations between local hyperalgesia and enhanced connectivity between the left superior parietal cortex and the left and right precentral gyrus were found.

**Conclusions:**

In line with our hypotheses, patients with CWAD showed the most pronounced alterations in resting-state functional connectivity, encompassing subcortical limbic (amygdala) and basal ganglia (pallidum), and ventral frontal regions (frontal operculum, orbitofrontal cortex) when compared to CINP and controls. Findings are generally in line with the idea of a continuum, in absence of significant group differences across CINP and CWAD. Enhanced amygdala-frontal operculum functional connectivity was the most robust and only connectivity pair in the cluster that was associated with QST (i.e., dynamic QST; endogenous pain inhibition), and that was observed in both patient groups. In addition, independent of group differences, enhanced resting-state functional connectivity between superior parietal cortex (involved in attention) and primary motor cortex was associated with static QST (i.e., greater local hyperalgesia). Taken together, our findings show a key role for enhanced amygdala-ventral frontal circuitry in chronic neck pain, and its association with diminished endogenous pain inhibition further emphasizes the link between cognitive-affective and sensory modulations of pain in women with chronic non-traumatic and traumatic neck pain.

## Highlights

• Amygdala-ventral frontal connectivity is enhanced in chronic neck pain.
• Enhanced amydala-frontal operculum coupling is related to decreased pain inhibition.
• Local hyperalgesia is associated with superior parietal-motor cortex connectivity.
• Symptoms and connectivity findings are most pronounced in traumatic neck pain.

## 1 Introduction

Chronic neck pain is worldwide a leading cause of disability with tremendous personal as well as socioeconomic impact (Fejer et al., 2006, Global, regional, and national incidence, prevalence, and years lived with disability for 328 diseases and injuries for 195 countries, 2016). Although there is clear overlap in signs and symptoms between chronic neck pain of a non-traumatic nature (i.e., chronic idiopathic neck pain, CINP) and traumatic origin (i.e., chronic whiplash associated disorders, CWAD) (Dimitriadis et al., 2015, Coppieters et al., 2017a, De Pauw et al., 2018, Sjolander et al., 2008), accumulating evidence demonstrates poorer outcomes in people with CWAD compared to CINP (Coppieters et al., 2017a, De Pauw et al., 2018, Ris et al., 2017, Scott et al., 2005). In CWAD, research has shown widespread hyperalgesia for various stimuli (i.e., increased pain sensitivity to e.g. pressure and heat), decreased endogenous analgesia (i.e., poorer functioning of descending inhibitory pathways of the central nervous system, as e.g. assessed using conditioned pain modulation/CPM), and enhanced nociceptive facilitation, including increased temporal summation of pain (i.e., increased efficacy of nociceptive signaling to the brain via ascending central nervous system pathways) compared to healthy pain-free controls (Coppieters et al., 2017a, Van Oosterwijck et al., 2013, Woolf, 2011, Stone et al., 2013). These sensory signs are indicative of nociplastic pain, which is ‘pain that arises from altered nociception despite no clear evidence of actual or threatened tissue damage causing the activation of peripheral nociceptors or evidence for disease or lesion of the somatosensory system causing the pain’ and of central sensitization (CS), which is an amplification of neural signaling within the central nervous system that elicits pain hypersensitivity (Woolf, 2011, den Boer et al., 2019, Trouvin and Perrot, 2019). In contrast, CS does not seem to be a typical feature of CINP. On a group-level, several studies have reported no evidence for widespread hyperalgesia and no decreased efficacy of CPM in patients with CINP compared to healthy pain-free controls (Coppieters et al., 2017a, Scott et al., 2005, Malfliet et al., 2015), although in subgroups and hence on an individual level, widespread hyperalgesia has been observed in individuals with CINP as well (Castaldo et al., 2018, Castaldo et al., 2019, Lopez-de-Uralde-Villanueva et al., 2016, Malfliet et al., 2015, Pina-Pozo et al., 2019, Walton et al., 2017). And, more recently, a meta-analysis revealed moderate-quality evidence of mechanical widespread hyperalgesia in patients with non-traumatic neck pain compared to pain-free controls (Xie et al., 2020). Thus, it is clear that interindividual differences in pain sensitivity are present in both chronic neck pain conditions (Walton et al., 2017, Castaldo et al., 2018), which may be best conceptualized as a continuum with CWAD on a further end of the spectrum compared to CINP. It is clear though that a better understanding of these sensory signs is needed, as signs of CS, as assessed with quantitative sensory testing (QST), are also present in various other chronic musculoskeletal pain disorders, and are generally associated with more (widespread) pain and disability, and poorer treatment responses (Nijs et al., 2019, Jull et al., 2007, Gerhardt et al., 2016, Uddin and MacDermid, 2016, Staud et al., 2012). Additionally, pain hypersensitivity is a possible risk factor for chronic pain and disability (Walton et al., 2011, Sarrami et al., 2017, Treede, 2019, Georgopoulos et al., 2019).

In line with increased pain sensitivity as assessed with QST, Magnetic Resonance Imaging (MRI) research has shown structural and functional brain changes in patients with chronic pain in regions associated with pain modulation (e.g., in descending nociceptive modulatory regions) (Tracey, 2010, Millan, 2002, Kregel et al., 2015, Ng et al., 2018, Apkarian, 2011, Cagnie et al., 2014, Smallwood et al., 2013, Davis and Moayedi, 2013, Coppieters et al., 2017b, Coppieters et al., 2018). Such brain changes have furthermore been related to levels of pain intensity (Coppieters et al., 2016), disability (De Pauw et al., 2019a), and pressure pain sensitivity (Coppieters et al., 2016, Niddam et al., 2016). In chronic neck pain specifically, our group has revealed alterations in grey matter morphology (i.e., both increases and decreases in volume and/or thickness) in women with chronic neck pain compared to healthy pain-free persons, in the precuneus, superior parietal cortex, precentral gyrus, superior temporal gyrus, and lateral orbitofrontal cortex, as well as white matter structural deficits in the cingulum hippocampus and tapetum (Coppieters et al., 2017b, Coppieters et al., 2018, De Pauw et al., 2019b). Furthermore, we observed associations between structural brain characteristics and impaired CPM and more pain and self-reported CS-related symptoms (e.g., in the precentral and postcentral gyrus, frontal orbital cortex, rostral middle frontal cortex, anterior and posterior cingulate cortex, thalamus, pars orbitalis, amygdala, insula, precuneus) (Coppieters et al., 2017b, Coppieters et al., 2018, De Pauw et al., 2019b). In addition, we have demonstrated changes in functional network properties during rest in the amygdala, posterior cingulate cortex, pallidum, and temporal pole in individuals with chronic neck pain compared to healthy controls using a graph theoretical approach (De Pauw et al., 2019a). Recently, another resting-state functional MRI (rs-fMRI) study observed multidirectional connectivity changes in the dorsolateral prefrontal cortex, hippocampus, dorsal posterior cingulate cortex, frontal pole, middle cingulate cortex, and anterior insula when comparing chronic neck pain patients with healthy pain-free persons (Ihara et al., 2019). The dorsolateral prefrontal cortex was a core hub of these altered functional networks. Thus, a whole array of regions has been identified to be of interest in patients with chronic neck pain, although rs-fMRI studies are scarce and associations between rs-fMRI and QST have not been investigated yet. In patients with chronic pain more broadly, altered resting-state functional connectivity (rsFC) (i.e., both increased and decreased rsFC) has been demonstrated in regions implicated in (chronic) pain, which has been associated with higher pain intensity and pain sensitivity, and decreased CPM efficacy (Coppieters et al., 2016, van Ettinger-Veenstra et al., 2019, Harper et al., 2018). It is unclear, however, whether these findings will extend to chronic neck pain, and whether there are differences across neck pain of non-traumatic versus traumatic nature in a way that is potentially related to QST measures.

The first aim is therefore to examine seed-to-seed rsFC alterations using predefined seeds in patients with CINP and CWAD compared to pain-free individuals and to investigate post hoc associations between rsFC group differences and self-reported pain-related and CS-related symptoms, and QST measures. The second aim is to investigate associations between seed-to-seed rsFC across all selected regions and self-reported CS-related symptoms, and QST measures. Brain regions in which we previously identified structural brain alterations or altered network properties based on resting-state fMRI graph theoretical analyses in this chronic neck pain sample were selected as seeds (Coppieters et al., 2017b, Coppieters et al., 2018, De Pauw et al., 2019a, De Pauw et al., 2019b), in addition to regions in which multidirectional rsFC changes were observed in another chronic neck pain sample (Ihara et al., 2019), and regions showing associations between structural or functional brain characteristics and pain-related measures in patients with chronic neck pain. It is hypothesized that 1) rsFC across selected seeds differs between pain-free controls and both patient groups, with more pronounced differences in patients with CWAD, and that these rsFC group differences are associated with self-reported symptoms and QST measures (across both groups, in line with a hypothesized continuum of symptoms and rsFC alterations across the neck pain groups), and 2) rsFC investigated across predefined regions (n = 22) is associated with CS-related symptoms, and QST measures indicative of pain hypersensitivity (across the entire patient group, in line with a continuum).

## 2 Materials and methods

### 2.1 Study design and procedure

This cross-sectional case-control study took place at the Department of Rehabilitation Sciences and Physiotherapy of Ghent University in cooperation with the Ghent Institute for Functional and Metabolic Imaging (GIfMI). Inclusion of participants took place from February 2014 until September 2015 and was carried out in accordance with the principles of the Declaration of Helsinki. The local Ethics Committee of the Ghent University Hospital (EC/2013/1053) approved the research protocol. All participants were thoroughly informed about the study procedures and signed an informed consent prior to study enrolment.

First, all participants completed a survey to acquire information on demographics, and filled out a series of questionnaires to obtain information on disability, pain intensity, and CS-related symptoms. Subsequently, QST assessments were performed. On a separate test day (10 ± 7 days apart), high-resolution T1-weighted magnetic resonance (MR) images, axial T2*-weighted images, diffusion-weighted images and resting-state functional MR images of the brain were acquired. Two researchers (IC, RDP) acquired the MRI scans and 1 researcher (IC) carried out the QST. Part of this dataset has already been described, with regard to alterations in grey matter morphology (Coppieters et al., 2017b, Coppieters et al., 2018), white matter structure (Coppieters et al., 2018), brain network properties using a graph theoretical approach (De Pauw et al., 2019a), and clinical behavioral differences between women with CINP and CWAD compared to controls (Coppieters et al., 2017a). The resting-state fMRI in combination with the QST, as presented here, has not been analyzed or described before.

### 2.2 Participants

107 female participants −38 patients with CINP, 37 patients with CWAD and 32 healthy pain-free controls– were enrolled in this study. In order to exclude the confounding factor of sex, only women were included, as research has demonstrated significant differences between men and women regarding functional brain alterations in chronic pain (Gupta et al., 2017), and pain sensitivity and pain processing in both healthy individuals and patients with chronic pain (Maleki et al., 2012, Bulls et al., 2015, Popescu et al., 2010). All participants were asked to stop intake of non-opioid analgesics 48 h prior to study participation. In addition, participants were asked not to undertake heavy physical exertion, and to refrain from consuming alcohol, caffeine and nicotine on the day of testing. All participants were Dutch native speakers and aged between 18 and 65 years. Participants were recruited by calls on social media and through advertisements placed on the Ghent University website, in health magazines, and in an information brochure of a whiplash patients association. Furthermore, flyers and posters were distributed in different medical institutes and associations in Flanders (hospitals, physiotherapy and medical physician practices).

Inclusion criteria for patients with CINP and CWAD were persistent neck pain lasting more than 3 months (Breivik et al., 2006), with a mean pain intensity of more than 3 out of 10 on the Visual Analogue Scale (VAS) during the past month. All chronic neck pain patients had to report mild/moderate to severe pain-related disability, established by a score of 10 or more out of 50 on the Neck Disability Index (NDI) (Vernon, 2008). Additionally, patients had to report stability of pain medication intake for at least 4 weeks prior to study participation. Inclusion criteria for patients with CINP were persistent idiopathic (non-traumatic) neck pain, with whiplash trauma being an exclusion criterion. Furthermore, patients with a specific cause of neck pain, e.g., cervical hernia with clinical symptoms, were excluded. Patients with CWAD were only included if they had neck pain resulting from a motor vehicle crash or traumatic event classifiable as WAD II A, B or C on the modified (Sterling, 2004) Quebec Task Force Scale (Spitzer et al., 1976).

Healthy pain-free women could participate if they were pain-free on each test day (VAS < 2/10)^1^, had no history of neck-shoulder-arm pain for longer than 8 consecutive days during the last year with a pain intensity of 2 or more out of 10 on the VAS, no medical consultation for neck-shoulder-arm pain during the last year and no history of a whiplash trauma. Additionally, healthy controls were only included if they had a score of <8 out of 50 on the NDI.

General exclusion criteria for all study groups were the presence of major depression, anxiety, psychiatric, neurologic, metabolic, cardiovascular and inflammatory disorders, fibromyalgia, chronic fatigue syndrome, and a history of neck or shoulder girdle surgery. Furthermore, all participants completed the MRI safety checklist and women who presented contraindications for MRI such as metal implants, a cardiac pacemaker, or pregnancy were excluded.

### 2.3 Self-reported measures

#### 2.3.1 Self-reported pain measures

On each test day, participants scored current neck pain intensity on the 11-point verbal numeric rating scale (VNRS-11). Scores range from 0 to 10, with 0 reflecting ‘no pain at all’ and 10 reflecting ‘the worst pain imaginable’. The validity of the VNRS-11 has been demonstrated (Ferreira-Valente et al., 2011). In addition, patients reported frequency of neck pain complaints in days per week and reported neck pain duration in months.

#### 2.3.2 Self-reported pain-related disability

The Dutch NDI was used to investigate self-reported pain-related disability levels (0–50) (Vernon, 2008, Vernon and Mior, 1991). Higher NDI scores indicate higher levels of pain-related disability. The Dutch NDI is proven to be reliable and valid to assess self-reported disability in patients with chronic neck pain (Ailliet et al., 2015, Jorritsma et al., 2010, Jorritsma et al., 2012).

#### 2.3.3 Self-reported symptoms related to central sensitization

All participants completed the Dutch version of the Central Sensitization Inventory (CSI). The CSI is a self-reported screening instrument to measure symptoms related to CS in chronic pain populations (Kregel et al., 2015, Mayer et al., 2012). The CSI part A assesses 25 overlapping somatic and emotional health-related symptom dimensions that have been reported to be associated with CS-related disorders (Cuesta-Vargas et al., 2018). Responses are recorded about the frequency of each symptom, with a Likert scale from 0 (never) to 4 (always), resulting in a total possible score of 100. Higher CSI scores denote a higher degree of self-reported CS-related symptoms and a recent systematic review found that the CSI generates valid and reliable data to quantify the severity of CS-related symptoms (Scerbo et al., 2018). Importantly, the CSI does not directly measure CS but it has been reported to correlate significantly with CS-related clinical variables such as higher pain intensity (Neblett et al., 2017) and wider pain distribution (van Wilgen et al., 2018). The Dutch CSI has good internal consistency, excellent test–retest reliability, and good discriminative power to differentiate between healthy individuals and chronic pain patients (Kregel et al., 2015).

### 2.4 QST measures

QST is able to measure pain processing changes with a psychophysical testing approach where the stimulus is quantified and used to measure perception and pain thresholds (static QST measures), and descending pain modulation (dynamic QST measures) (Mucke et al., 2016, Uddin and MacDermid, 2016).

#### 2.4.1 Local (primary) and distant (widespread) pressure hyperalgesia

The pressure pain threshold was measured unilaterally with a digital pressure algometer with a 1 cm^2^ tip (Wagner Instruments, FDXTM, Greenwich) at a symptomatic local region (middle trapezius muscle midway between the spinous process of C7 and the lateral border of the acromion) to assess local (primary) pressure hyperalgesia (Coppieters et al., 2017a). In addition, the pressure pain threshold was measured unilaterally with the digital pressure algometer at a distant asymptomatic region (quadriceps muscle midway between the SIAS and the basis patellae) to evaluate distant or widespread pressure hyperalgesia (Meeus et al., 2010, Whiteside et al., 2004).

The pressure pain thresholds were assessed at the most painful side (Kosek et al., 1999). In healthy women, and when patients experienced the same amount of neck pain at both sides, the pressure pain thresholds were tested at the dominant side. The participant was comfortably seated, and pressure was gradually increased at a rate of 1 kg/s until the participant reported the first sensation of unpleasantness. The pressure pain threshold was determined as the mean of 2 consecutive (30 s in between) measurements. This technique was found to be reliable (Cathcart and Pritchard, 2006). In addition, the intratester reliability of the pressure pain threshold measurements is satisfactory to good (ICC 0.78–0.93) (Ylinen et al., 2007).

#### 2.4.2 Efficacy of conditioned pain modulation

The presence of dysfunctional endogenous pain inhibition was investigated by evaluating the efficacy of CPM by applying a CPM paradigm. This paradigm relies on the 'pain-inhibits-pain' mechanism in which one noxious stimulus is used as a conditioning stimulus to induce reduction in pain perception of another test stimulus (Yarnitsky, 2010). The conditioning stimulus for eliciting CPM was the cold pressor test. The pressure pain threshold assessment was used as test stimulus. Then the hand was immersed in water maintained at room temperature (22 °C) for 1 min to standardize the hand temperature (Ng et al., 2014). Subsequently, the participant was asked to immerse the same hand (up to the wrist) in a refrigerated circulating bath (VersacoolTM, Thermo Fisher Scientific, Newington NH, USA) with cold water maintained at 12 ± 1 °C (Tousignant-Laflamme et al., 2008). The contralateral hand to the test stimulus was used to maximize the CPM effect, which is dependent on the distance between both stimuli (Rehberg et al., 2012). The participants held their hand in the water bath for a period of 2 min (Ng et al., 2014). The pressure pain threshold was re-evaluated at the previously defined location (quadriceps muscle). This re-evaluation started at 45 s after immersing the hand, and the second pressure pain threshold measurement was performed with at least 30 s in between (Lewis et al., 2012). When the participants removed their hand out of the water before the end of the 2 min, the measurement was registered as missing. In order to analyze CPM efficacy, the mean pressure pain threshold measured during the cold pressor test was subtracted from the mean pressure pain threshold measured before the cold pressor test. Hence, a negative CPM value indicates a normal CPM response (i.e., endogenous pain inhibition) and a positive CPM value reflects an abnormal CPM response (i.e., pain facilitation) with a higher positive CPM value reflecting less efficient endogenous pain inhibition and more pain facilitation (Yarnitsky et al., 2015). The intrasession intraclass correlation coefficient for the cold pressor test is excellent (0.85) (Lewis et al., 2012). A gold standard for CPM testing is not available, but pressure pain thresholds and cold water immersion are the most commonly used test and conditioned stimuli, respectively (Kennedy et al., 2016).

### 2.5 MRI data acquisition

Magnetic Resonance images were acquired on a 3 T Siemens Magnetom TrioTim MRI scanner (Siemens, Erlangen, Germany) equipped with a 32-channel matrix head coil, located at GIfMI, Ghent University Hospital, Ghent, Belgium. High-resolution whole-brain T1-weighted MR images of the brain were acquired using a three-dimensional magnetization prepared rapid acquisition gradient echo sequence (MP-RAGE) with the following parameters, repetition time (TR) = 2250 ms, echo time (TE) = 4.18 ms, voxel size = 1 × 1 × 1 mm, FoV-matrix = 256 × 256 mm, flip angle = 9°, 176 coronal slices, acquisition time (TA) = 5′14′′;. All T1-weighted anatomical scans were checked to assure that no brain lesions were present. Axial T2*-weighted brain images were acquired using a T2*-weighted acquisition gradient echo with TR = 893 ms, TE = 18.6 ms, voxel size = 1 × 0.7 × 3 mm, FoV read = 230 × 230 mm, flip angle = 20°, 33 slices, and an acquisition time of 3′ 48′′. All T2*-weighted images were visually inspected by 2 expert neuroradiologists (KD, EG) to evaluate possible microhemorrhages or hemorrhagic shearing lesions related to trauma or diffuse axonal injury. No hemorrhagic shearing lesions or microhemorrhages related to trauma or axonal injury were detected.

Resting-state fMRI was acquired using a T2*-weighted EPI sequence with the following parameters: TR = 2000 ms, TE = 29 ms, flip angle = 90°, number of slices = 40, FoV read = 192 mm × 192 mm, voxel size = 3 × 3 × 3 mm, 300 volumes, and acquisition time = 10′12′′. Participants were asked to close their eyes but to remain awake and not fall asleep. In addition, they were asked not to think about anything in particular. None of the participants reported falling asleep during the resting-state fMRI scanning procedure.

### 2.6 Data analyses

#### 2.6.1 Behavioral data

Normality of variables was examined with the Shapiro-Wilk test and by visual evaluation of histograms and quantile–quantile plots. The Levene’s test was used to assess the equality of variance. If the assumptions of normality and equal between-group variances were met, data were analyzed with parametric tests. Otherwise, nonparametric tests were applied. Group differences in demographics, self-reported pain-related and CS-related outcomes and QST variables were investigated with a one-way ANOVA with post-hoc pairwise comparisons or with the Kruskal-Wallis test with post-hoc pairwise comparisons using the Mann-Whitney *U* test. All statistical analyses of the behavioral data were performed with IBM SPSS Statistics version 26 at a significance level of α = 0.05. Bonferroni correction was applied where necessary to correct for the number of post-hoc comparisons.

In addition to the CPM effect being analyzed as a continuous variable, CPM was also considered as a categorical variable to calculate the percentage of participants in each group showing an inhibitory (normal) versus facilitatory (abnormal) CPM effect. A Chi-Square test was performed to analyze group differences.

#### 2.6.2 MRI data preprocessing

MRI data were analyzed using CONN (Whitfield-Gabrieli and Nieto-Castanon, 2012). For resting-state functional data, the first 4 volumes of each complete time series were discarded because of saturation effects. Preprocessing included correction for slice time differences, 3D head motion correction, segmentation into white matter, grey matter and cerebral spinal fluid (DARTEL), normalization into Montreal Neurological Institute (MNI) space, and spatial smoothing (Gaussian filter FWHM of 6 mm). A visual data quality check was performed by two researchers (IC, IT) including raw data, normalization and plots of the motion parameters.

#### 2.6.3 Motion

Motion parameters were inspected. Participants were excluded if any of the six absolute motion parameters exceeded 2 mm/degrees and/or if the mean framewise displacement exceeded 0.2 mm (Parkes et al., 2018). Based on these motion thresholds, 7 participants were excluded including 2 healthy participants, 1 patient with CINP, and 4 patients with CWAD. The final MRI sample therefore consisted of 100 participants, including 37 patients with CINP, 33 patients with CWAD and 30 healthy pain-free controls.

Furthermore, mean absolute and maximum absolute motion across the six parameters, mean and maximum framewise displacement, number of outliers volumes (identified by ART; see 2.6.4), and mean and maximum global BOLD signal changes were compared between the groups. There were no significant group differences in mean absolute motion (*F*_2,97_ = 1.37, *p* = .26), maximum absolute motion (*F*_2,97_ = 0.67, *p* = .51), mean framewise displacement (*F*_2,97_ = 1.13, *p* = .33), maximum framewise displacement (*F*_2,97_ = 1.02, *p* = .36), number of outliers volumes (F_2,97_ = 1.09, p = .34), mean global BOLD signal changes (*F*_2,97_ = 0.66, *p* = .52), and maximum global BOLD signal changes (*F*_2,97_ = 0.23, *p* = .79) (see Figure S1).

#### 2.6.4 Denoising and first-level analysis

For denoising purposes, the six absolute motion parameters and their first derivatives were added as regressors of no interest, as well as noise signals from the cerebral spinal fluid and white matter (estimated using anatomical component-based noise correction, aCompCor; 10 parameters). Denoising also included scrubbing of excessive motion by means of ART outlier detection (% volumes removed: mean = 0.32%, range = 0.00–6.00%; Figure S1). Outlier time points are identified via the framewise motion displacement parameters and global signal intensity using ART (nitrc.org/projects/artifactdetect). For each participant, we treated images (time points) as outliers if composite movement from a preceding image exceeded 0.9 mm (framewise displacement, as defined by ART), or if the global mean intensity was greater than 5 standard deviations from the mean image intensity for the entire resting scan. A variable number of outlier regressors (i.e., one for each identified outlier time point) was then included as regressors of no interest to remove any influence of these outlier scans on the BOLD signal (Nieto-Castanon, 2020). Furthermore, linear detrending was performed and signal oscillations at a frequency of 0.005–0.1 Hz (simultaneous) were added as confounders for band-pass filtering of the time series. The first-level analysis then estimated bivariate correlation coefficients between the defined regions of interest (ROIs).

#### 2.6.5 Regions of interest (ROIs)

Guided by previous findings regarding brain alterations in patients with chronic neck pain, ROI-to-ROI (Whitfield-Gabrieli and Nieto-Castanon, 2012) rsFC analyses were conducted with the following seed regions: precuneus, posterior cingulate cortex, left and right insula, left and right amygdala, anterior cingulate cortex, medial prefrontal cortex, left and right hippocampus, left and right thalamus, left and right pallidum, left and right temporal pole, left and right superior parietal cortex, left and right precentral gyrus, left and right superior temporal gyrus anterior and posterior division, left and right supramarginal gyrus anterior and posterior division, left and right frontal operculum, left and right middle frontal gyrus, left and right frontal orbital cortex, left and right postcentral gyrus, left and right superior frontal gyrus, and left and right frontal pole. These seeds (i.e., 22 seeds bilateral; 40 seeds when taking left and right separate) are observed to be structurally or functionally altered in previous studies in the chronic neck pain population of the present study (Coppieters et al., 2017b, Coppieters et al., 2018, De Pauw et al., 2019a, De Pauw et al., 2019b) or in another chronic neck pain sample (Ihara et al., 2019) compared to healthy controls. Also, seeds were selected based on our previously observed significant associations between structural or functional brain characteristics and pain-related measures in CINP and CWAD. All regions were extracted from the Harvard-Oxford cortical and subcortical atlas thresholded at 0.25.

#### 2.6.6 Second-level analysis

ROI-to-ROI (i.e., seed-to-seed) rsFC analyses were conducted in the CONN toolbox v20b using the recommended settings for cluster-based inferences: parametric multivariate statistics (Whitfield-Gabrieli and Nieto-Castanon, 2012). Potential rsFC group differences were examined between defined seeds. Age and mean framewise displacement were included as covariates in all analyses. For the main effect of group, functional network connectivity with a FDR-corrected *p* < .05 cluster-level threshold together with a post-hoc uncorrected *p* < .05 height (connection-level) threshold was applied, which is a standard and appropriate choice for thresholding ROI-to-ROI parametric maps while appropriately controlling the family-wise error rate (Nieto-Castanon, 2020). Bivariate correlations were extracted for rsFC pairs showing significant main effects of group, transformed using Fisher's z, and post-hoc pairwise group comparisons were performed in IBM SPSS version 26 (using a Bonferroni correction). In addition, within these rsFC pairs showing group differences, post hoc associations with disability, pain intensity, self-reported CS-related symptoms, and static and dynamic QST measures were examined within the patient group (CINP and CWAD together) using Pearson correlations. Post-hoc, we inspected whether there were any differences in correlations across groups, by means of an interaction effect. In specific, a linear regression was run with the rsFC as dependent variable, and the QST measure and the patient group variable (including CINP and CWAD) as predictors in the first model, while adding the product term of the patient group variable and the QST measure in the second model. Change in explained variance (R^2^) across models was inspected, as well as the interaction term.

In addition, within the patient group (taking both groups together), associations were explored in CONN between rsFC across all selected seeds and self-reported symptoms related to CS (i.e., CSI scores), and static (PPT at a local and distal location) and dynamic (CPM) QST measures (using the same FDR-corrected *p* < .05 cluster-level threshold with a post-hoc uncorrected *p* < .05 connection-level threshold). For significant associations, bivariate correlations were extracted, transformed using Fisher’s z and visually plotted. Again, we inspected whether there were any differences in correlations across groups by assessing a potential interaction effect.

## 3 Results

### 3.1 Demographic characteristics, pain-related outcomes, self-reported CS-related symptoms and QST measures

The demographic characteristics, pain duration and frequency of 107 female participants (38 patients with CINP, 37 patients with CWAD and 32 healthy pain-free controls) are presented in Table 1. No significant differences in demographic characteristics were observed between the groups, except for age (healthy controls were younger compared to CINP and CWAD patients). Both neck pain groups were comparable in terms of neck pain duration but CWAD patients reported a higher frequency of neck pain complaints per week compared to CINP patients.

**Table 1:** Demographic characteristics and self-reported pain measures in patients with CWAD, CINP and healthy controls.

			Median	IQR	Range (min–max)	Test statistic (*P*-value)	*P*-value post-hoc
***Demographic characteristics***	**Age (y)**^a^	**HCON**	24	22–37,75	18–62	10.784 (**0.005**)	**0.006**^c^ **0.003**^d^ 0.966^e^
		**CINP**	36	27,75–47,00	18–62		
		**CWAD**	38	25,50–47,50	21–59		
	**Body mass Index (kg/m^2^)**^a^***^,^***^†^	**HCON**	21,08	20,20–22,77	17,99–26,75	2.385 (0.303)	0.127^c^ 0.593^d^ 0.314^e^
		**CINP**	23,03	20,02–25,36	18,34–26,75		
		**CWAD**	22,10	19,51–23,97	16,65–31,46		
			**Frequencies**				
	**Handedness, n (%)**^b^ (LH; RH)	**HCON**	5 (15.63); 27 (84.38)			2.686 (0.282)	NA
		**CINP**	2 (5.26); 36 (94.74)				
		**CWAD**	2 (5.41); 35 (94.59)				
*Demographic characteristics*: regular medication use*	**Analgesics – antipyretics, n (%)**^b^	**HCON**	0 (0)			12.211 (**0.001**)	0.036^e^
		**CINP**	3 (7.9)				
		**CWAD**	10 (27)				
	**Narcotic analgesics, n (%)**^b^	**HCON**	0 (0)			2.564 (0.205)	NA
		**CINP**	0 (0)				
		**CWAD**	2 (5.4)				
	**Antidepressants, n (%)**^b^	**HCON**	0 (0)			2.386 (0.371)	NA
		**CINP**	3 (7.89)				
		**CWAD**	2 (5.4)				
			**Median**	**IQR**	**Range (min**–**max)**	**Test statistic** **(*P*-value)**	***P*-value post-hoc**
***Self-reported pain measures***	**Neck pain duration (months)**^a^	**HCON**	NA	NA	NA	559.500 (0.670)	NA
		**CINP**	60	23,50–120	4–288		
		**CWAD**	60	30–120	3–444		
	**Days/week neck pain**^a^	**HCON**	NA	NA	NA	239.500 (**0.022**)	NA
		**CINP**	5	3.75–7	3–7		
		**CWAD**	7	5–7	2–7		

a = Data which were not normally distributed and subsequently group differences were analyzed using the Kruskal-Wallis test and for post-hoc pairwise comparisons the Mann-Whitney *U* test. To correct for multiple comparisons, differences measured with the Mann-Whitney *U* test were only deemed significant below the 0.017 level (Bonferroni correction: 0.05/3).

b = categorical data were analyzed by performing the Fisher’s exact test (post-hoc pairwise comparisons were only deemed significant below the 0.017 level). Significant differences are presented in Bold. ^†^Variances were not equally distributed across groups, Levene’s test *p* < 0.05.

c = *p*-value for differences between HCON-CINP.

d = *p*-value for differences between HCON-CWAD.

e = *p*-value for differences between CINP-CWAD.

* Participants were asked to refrain from the intake of non-opioid analgesics 48 h before testing. y = years. HCON = healthy pain-free controls, CWAD = chronic whiplash-associated disorders, CINP = chronic idiopathic neck pain. IQR = interquartile range, n = number.

**Fig. 1:**
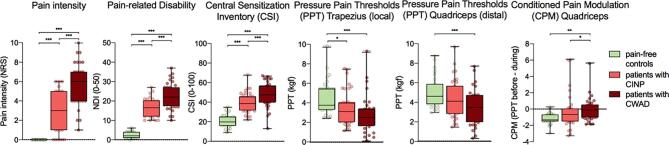
Self-reported pain-related outcomes (n = 100), self-reported symptoms related to CS (n = 100), and QST measures (n = 98) across groups. Box plots and values of individual participants are presented. Significant values reflect results of post-hoc pairwise comparisons of one-way ANOVA or Kruskal-Wallis test. Main effects of group are not depicted for visual simplicity. All *p* values are Bonferroni corrected. *= *p* < .05, **= *p* < .01, ***= *p* < .005. CS: central sensitization, QST: quantitative sensory testing, NDI: Neck Disability Index.

Figure 1 presents the pain-related outcomes, self-reported symptoms related to CS, and QST measures per group. A significant main effect of group was present for pain intensity (*H*_2_ = 68.79, *p* < .001), pain-related disability (*H*_2_ = 68.81, *p* < .001), and CSI scores (*H*_2_ = 57.91, *p* < .001). Patients with CWAD reported higher neck pain intensity on the day of MRI testing (*p* < .001. Cohen's d = 1.37) and more severe neck pain-related disability (*p* = .001, Cohen's d = 1.00) compared to the CINP group. Patients with CWAD and CINP reported significantly more symptoms associated with CS than healthy women (both *p* < .001; Cohen's d = 2.77, Cohen's d = 2.28, respectively), and women with CWAD reported significantly more CS-related symptoms compared to CINP (*p* = .001, Cohen's d = 0.75). Also, a significant main effect of group was found for local hyperalgesia (*H*_2_ = 15.01, *p* = .001), distant hyperalgesia (*F*_2,94_ = 5.85, *p* = .004), and CPM effect (*F*_2,84_ = 6.04, *p* = .004). Both patient groups showed significantly decreased pressure pain thresholds at the trapezius muscle (i.e., local hyperalgesia) compared to healthy controls, while the patient groups did not differ (CWAD-controls: *p* < .001, Cohen's d = 0.87; CINP-controls: *p* = .010, Cohen's d = 0.56; CINP-CWAD: *p* = .14). Furthermore, patients with CWAD showed significantly decreased pressure pain thresholds at the quadriceps muscle (i.e., distant hyperalgesia) compared to healthy controls (*p* = .003, Cohen's d = 0.46), while there were no significant differences between CINP patients and healthy controls (*p* = .38) and CWAD patients (*p* = .15). In addition, efficacy of CPM was significantly lower in people with CWAD compared to CINP patients (*p* = .02, Cohen's d = 0.69) and healthy controls (*p* = .006, Cohen's d = 0.91). In contrast, individuals with CINP and healthy controls demonstrated no significant differences in CPM effect (*p* > .99). Also when looking at percentages of individuals showing normal versus abnormal CPM responses, it was observed that less patients with CWAD (n = 19, 65.5%) showed an inhibitory CPM effect compared to healthy controls (n = 25, 92.6%) (chi-square; *p* = .014) but no significant differences were found between patients with CINP (n = 25, 83.3%) and healthy controls or CWAD (chi-square; *p* = .29; *p* = .12, respectively). During the CPM test, 5 patients with CINP and 5 patients with CWAD removed their hand out of the cold water before the end of the 2 min because the test was too painful.

### 3.2 Group differences in rsFC

**Fig. 2:**
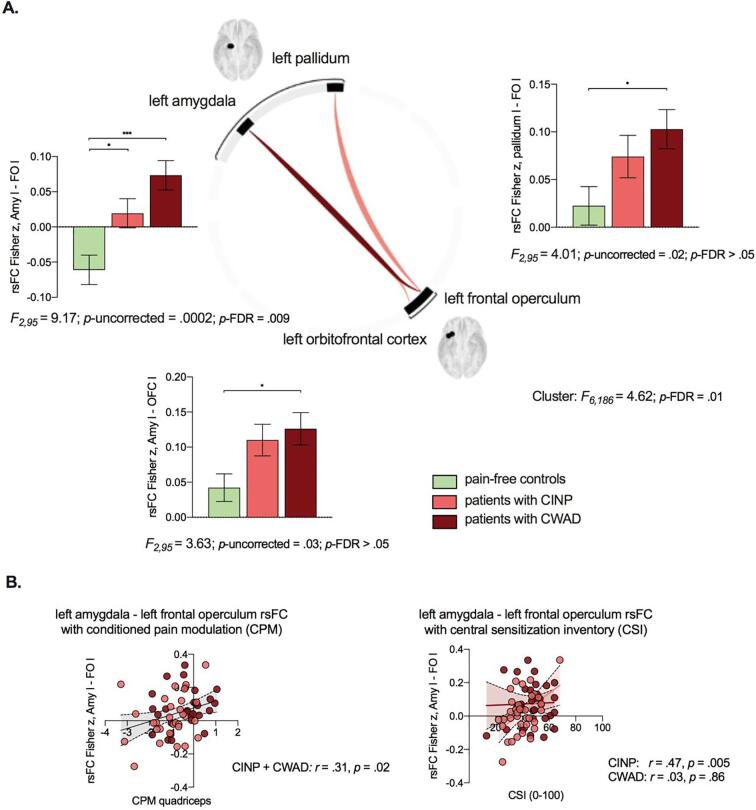
rsFC pairs showing a significant main effect of group (*p*-FDR < 0.05) (A). Statistics refer to the pairwise group comparisons. *= *p* < .05, ***= *p* < .001. *p* values are Bonferroni corrected. Amy: Amygdala, l: left, OFC: Orbitofrontal cortex, FO: frontal operculum, rsFC: resting-state functional connectivity. Scatterplots of associations between rsFC showing group differences and CPM (taking CINP and CWAD together), and for associations with CSI taking CINP and CWAD separately because of significant interaction effect with group (B).

One significant cluster was detected in the rsFC analysis in CONN (*F*_6,__186_ = 4.62, *p*-FDR = 0.01), comprising three rsFC pairs across the amygdala, pallidum, frontal operculum and frontal orbital cortex (Figure 2A). In specific, a significant main effect of group was observed in rsFC between the left amygdala and left frontal operculum, which also survived even more stringent connection-level *p*-FDR correction (*F*_2,95_ = 9.17, *p*-uncorrected = 0.0002, *p*-FDR = 0.009). As presented in Figure 2A, pairwise group comparisons revealed that both patients with CWAD and with CINP showed enhanced amygdala-frontal operculum rsFC compared to healthy controls (CWAD: *p* < .001, Cohen's d = 1.15; CINP: *p* = .02, Cohen's d = 0.67). No rsFC group differences were observed between patients with CWAD and CINP (*p* = .19). In addition, a main effect of group in rsFC between left amygdala and left frontal orbital cortex (*F*_2,95_ = 4.01, *p*-uncorrected = 0.02), and between left pallidum and left frontal operculum (*F*_2,95_ = 3.63, *p*-uncorrected = 0.03) was observed. The pairwise group comparisons demonstrated enhanced rsFC in patients with CWAD compared to healthy controls between the left amygdala and left frontal orbital cortex (*p* = .03, Cohen's d = 0.70), and between the left pallidum and the left frontal operculum (*p* = .03, Cohen's d = 0.70). No significant rsFC differences for these connections were revealed between patients with CINP and healthy controls, or between both patient groups (*p* > .05).

Zooming into these group differences by performing post-hoc associations, we observed that across all patients (taking CINP and CWAD together), enhanced rsFC between left amygdala and left frontal operculum was associated with more self-reported CS-related symptoms (*r* = 0.30, *p* = .02) and decreased efficacy of CPM (*r* = 0.31, *p* = .02) (Figure 2B). Follow-up analyses showed that the association with self-reported CS-related symptoms interacted with group (interaction term *β* = −1.09, *t* = −1.99, *p* = .05), and hence correlations are presented separately per group, emphasizing that this effect is driven by the CINP group (CINP: *r* = 0.47, *p* = .005; CWAD: *r* = 0.03, *p* = .86; see also Table S1). The association with CPM did not show an interaction with group (Table S1 and Figure S2A). For the amygdala-orbitofrontal and pallidum-frontal operculum rsFC, we observed no significant associations with self-reported symptoms and QST measures across all patients (*p* > .05).

### 3.3 Associations between rsFC across all selected regions and self-reported symptoms related to CS and QST measures

**Fig. 3:**
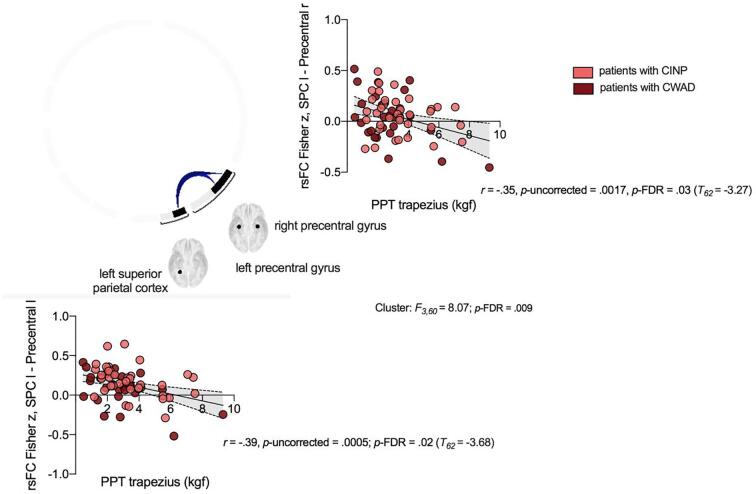
Scatterplots of associations between higher local pressure hyperalgesia (i.e., decreased PPTs), and increased rsFC between left superior parietal cortex and respectively right and left precentral gyrus at the level of all selected regions in patients with chronic whiplash associated disorders (CWAD) and chronic idiopathic neck pain (CINP). PPT: pressure pain threshold, rsFC: resting-state functional connectivity.

When exploring rsFC across all selected regions in the patient group specifically, we observed that lower pressure pain thresholds at the trapezius muscle (i.e., local hyperalgesia) were associated with enhanced rsFC in a cluster (*F*_3, 60_ = 8.07, *p*-FDR = 0.009) comprising of left superior parietal cortex and bilateral precentral gyrus (Figure 3). In specific, we observed that decreased pressure pain thresholds (i.e., higher local hyperalgesia) was associated with enhanced rsFC between the left superior parietal cortex and both right precentral gyrus (*r* = −0.35, *p*-uncorrected = 0.0017, *p*-FDR = 0.03) and left precentral gyrus (*r* = −0.39, *p*-uncorrected = 0.0005, *p*-FDR = 0.02) (Figure 3). Neither associations showed an interaction with group (Table S1 and Figure S2B). No other associations were identified.

## 4 Discussion

**Fig. 4:**
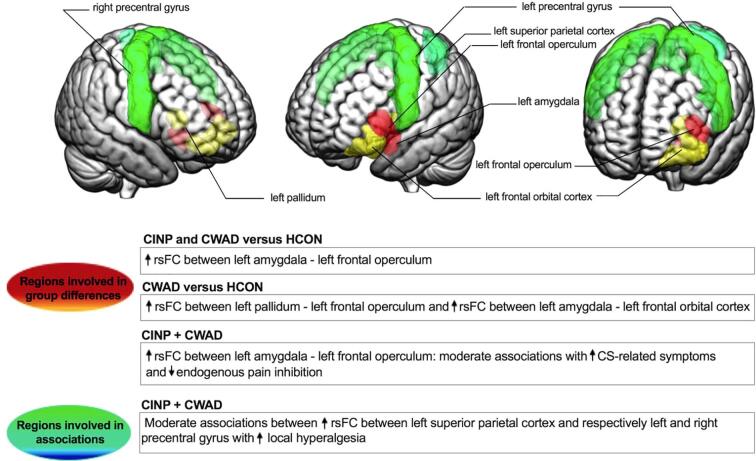
Overview of ROIs showing significant group differences in rsFC between CINP and CWAD patients and pain-free controls, and ROIs involved in significant associations between rsFC across all selected regions and local hyperalgesia within CINP and CWAD patients. ROIs: regions of interest, rsFC: resting-state functional connectivity, CS: central sensitization, CINP: chronic idiopathic neck pain, CWAD: chronic whiplash associated disorders.

In this study, seed-to-seed rsFC alterations were examined in women with CINP and CWAD compared to pain-free women, as well as associations between rsFC and self-reported pain-related and CS-related symptoms and QST measures within the patient group. The main findings (as summarized in Figure 4) were that 1) both patient groups showed enhanced left amygdala coupling with the left frontal operculum compared to controls, which was associated with self-reported CS-related symptoms (only in CINP) and a dynamic QST measure (i.e., CPM), 2) patients with CWAD furthermore showed enhanced rsFC between left pallidum and left frontal operculum, and between left amygdala and left frontal orbital cortex compared to controls, which was not related to QST measures, and 3) within the patient group, greater rsFC between the left superior parietal cortex and bilateral precentral gyrus was associated with higher local hyperalgesia. Trauma-induced CWAD and non-traumatic CINP did not show significant seed-to-seed rsFC differences compared to each other, although qualitatively patients with CWAD showed more extensive alterations compared to CINP (i.e., larger effect sizes in the difference with controls). To the best of our knowledge, this is the first study to reveal a key role for enhanced rsFC in amygdala-ventral frontal circuitry in patients with chronic neck pain, which is associated with altered sensory processing of pain, suggesting involvement of this circuitry in maintenance of pain hypersensitivity.

### 4.1 Self-reported symptoms and QST measures

Both chronic neck pain groups reported significant pain-related disability and symptoms related to CS, but patients with CWAD reported more severe problems compared to patients with CINP, as reported previously. A similar amount of local hyperalgesia was present across both patient groups, but only participants with CWAD demonstrated distant hyperalgesia and disturbed endogenous pain inhibition. On a group level, these results indicate that while peripheral sensitization is present in both CINP and CWAD patient groups, signs indicative of CS or nociplastic pain are only present in patients with CWAD (Graven-Nielsen and Arendt-Nielsen, 2010, Kosek et al., 2016). However, both patient groups show a wide range in severity of symptoms and in QST measures, and overlap across these groups exists. A small proportion of patients with CINP, for instance, also show abnormal CPM, although on a group-level CPM is not impaired. This is in line with a continuum, as opposed to clearly separable groupings across these chronic neck pain populations, with increasing severity of symptoms and alterations in pain processing. The idea of a continuum is in accordance with other research investigating patients with CINP and CWAD (Malfliet et al., 2015, Walton et al., 2017, Castaldo et al., 2019, Castaldo et al., 2018)*.*

### 4.2 Group differences in rsFC

The group differences in rsFC center around a cluster comprising of the amygdala and pallidum as subcortical regions, and frontal operculum and frontal orbital cortex as ventral regions in the frontal cortex (with frontal orbital cortex or orbitofrontal cortex lying more medial than the frontal operculum region). The most robust group difference was enhanced rsFC between amygdala and a region in the ventrolateral frontal cortex (left frontal operculum), which was observed both in women with CINP and CWAD, as compared to pain-free controls. Across the entire patient group, we observed that enhanced amygdala-frontal operculum coupling was moderately associated with decreased endogenous pain inhibition, and in CINP it was furthermore related to more self-reported CS-related symptoms. This rsFC pair was not related to pain intensity, disability, and hyperalgesia.

The amygdala, as part of the limbic system, is involved in affective (or more specifically threat) processing and regulation (Krabbe et al., 2018, LeDoux et al., 2003, Phelps and LeDoux, 2005) and contributes to affective-emotional components of persistent pain through connectivity with the prefrontal cortex (Simons et al., 2014). The ventrolateral prefrontal cortices have been associated with the generation and regulation of (unpleasant) emotions through connectivity with the amygdalae (Wager et al., 2008), with a proposed key role for perceived control and for reappraisal of emotional significance of aversive stimuli, including pain (Salomons et al., 2007, Wiech et al., 2008). However, the frontal opercular region is located more posterior, and hence although it can be argued to be part of the ventrolateral prefrontal cortex, it is important to note that it is anatomically more adjacent to (i.e., lies dorsal to) the insula (Maliia et al., 2018, Naidich et al., 2004)*.* A role of the frontal operculum and adjacent dorsal insula in the early sensory-discriminative dimensions of pain processing has been suggested (Schlereth et al., 2003), although it has shown associations with cognitive-affective dimensions of pain as well. For instance, an insular cluster (partly overlapping with frontal operculum) was found to be activated by sensory aspects of a nociceptive stimulus as well as by its perceived controllability (Salomons et al., 2004). Additionally, a recent *meta*-analysis of conditioning studies demonstrated that the frontal operculum is a robust neural correlate of pain-related fear (Biggs et al., 2020). This is particularly interesting given the enhanced rsFC with the amygdala that we found, and the putative role of amygdala in threat processing. Regardless of the precise spatial localization and bearing in mind the reverse inference problem (Poldrack, 2011), our findings show enhanced amygdala coupling with the frontal operculum, which is furthermore associated with altered endogenous pain inhibition in both chronic neck pain groups, but not with other (static) QST measures.

These finding are in line with previous studies. For instance, Ihara et al. recently also revealed aberrant prefrontal rsFC in patients with chronic neck pain compared to healthy controls, albeit in coupling of the dorsolateral prefrontal cortex (Ihara et al., 2019). Also in our previous graph analytical resting-state analysis, we found alterations in the amygdala (De Pauw et al., 2019a). In particular, higher levels of intramodular degree were observed in both chronic neck pain patient groups compared to healthy controls in the amygdala but also in the pallidum indicating differences in the functioning of brain hubs (i.e., regions that are responsible for orchestrating communication between other brain regions). Moreover, these changes were correlated with self-reported CS-related symptoms, disability and neuromuscular control. Furthermore, we found associations between decreased grey matter volume in the amygdala and more symptoms related to CS and cognitive deficits, and between decreased grey matter volume in the pars orbitalis (i.e., overlapping with frontal operculum) and pain catastrophizing and cognitive deficits in CWAD (Coppieters et al., 2017b). Enhanced amygdala rsFC has also been observed in several other chronic pain conditions, including enhanced coupling with central executive network in chronic low back pain (Jiang et al., 2016), and with several regions including the (ventrolateral) prefrontal cortex regions in pediatric patients with complex regional pain syndrome (Simons et al., 2014). These findings are generally in accordance with the suggested importance of corticolimbic connectivity, and increasing engagement of emotion and reward circuits (Hashmi et al., 2013) in the development and maintenance of chronic pain )(Vachon-Presseau et al., 2016). Our finding that aberrant fronto-limbic coupling during rest is associated with diminished endogenous pain inhibition emphasizes the link between cognitive-affective and sensory modulations of pain in patients with chronic pain. Also in other patient populations, including migraine and fibromyalgia, rsFC has been associated with endogenous pain inhibition (Argaman et al., 2020, Harper et al., 2018).

The other rsFC pairs (amygdala-orbitofrontal cortex, and pallidum-frontal operculum) were only significantly enhanced in patients with CWAD compared to controls (although there was a linear trend across CWAD, CINP and controls), and were not associated with any of the self-reported or QST measures. The amygdala is a core threat center and part of the limbic system and the pallidum is a critical node in the mesolimbic network (Wulff et al., 2019) and part of basal ganglia involved in movement and reward (Buot and Yelnik, 2012). Both are strongly interconnected to the frontal cortex (Buot and Yelnik, 2012). Also, pallidum and amygdala are both (in)directly involved in the selection process of the most appropriate motor response (Grèzes et al., 2014, Grillner et al., 2005). Furthermore, the ventral pallidum is an important output for limbic signals and is critically involved in encoding expected reward value and regulating motivated behaviors (Smith et al., 2009). Note that we did not use a subdivision for the pallidum, so further research will be needed to examine which subpart is specifically altered. The orbitofrontal cortex plays a key role in emotional processing, reward and decision-making (Rolls et al., 2020) and is part of the salience network (Bhatt et al., 2019).

In chronic low back pain, previous research showed perturbed connectivity of the amygdala with the default mode network, including also the medial prefrontal cortex (Jiang et al., 2016). We previously only observed decreased grey matter volume in the lateral orbitofrontal cortex in this sample for CWAD patients compared to healthy controls (albeit only in the right hemisphere) (Coppieters et al., 2017b), and we found associations between decreased grey matter volume in the lateral orbitofrontal cortex and cognitive deficits in CWAD patients. Here, we extend these findings showing that the functional connectivity of the orbitofrontal cortex with the amygdala is also altered.

Taken together, our rsFC results of enhanced coupling in circuitry comprising ventromedial fronto-limbic and ventrolateral frontal-pallidum rsFC suggest alterations that are potentially related to movement, reward, emotional processing and/or decision-making, in particular in patients with CWAD, going beyond pain sensitivity. Further research is warranted to examine whether these circuit alterations may be related to cognitive deficits, motor impairment, and stress that have been observed in patients with CWAD.

### 4.3 Associations between rsFC across all selected regions and self-reported symptoms related to CS and QST measures

In addition, associations between rsFC and CS-related symptoms and QST measures were explored in the patient group across all selected regions. Moderate correlations (*r* = −0.35 to *r* = −0.39) were identified between local hyperalgesia at the trapezius muscle (i.e., static QST) and enhanced rsFC between the left superior parietal cortex and bilateral primary motor cortex (i.e., M1, precentral gyrus) in patients with chronic neck pain. Noteworthy, these regions were not involved in the observed rsFC group differences. No additional associations with endogenous pain inhibition (i.e., CPM efficiency, or dynamic QST) or self-reported CS-related symptoms were observed. M1 is part of the sensorimotor network and has an essential role in motor execution (Chang et al., 2018). The superior parietal lobule is a heterogeneous region involved in various functional processes including cognitive processes such as attention and working memory but also somatosensory and visuomotor integration (Wang et al., 2015). This region is an important hub in the central executive resting-state network (Wang et al., 2020). Accordingly, our results suggest a role for altered rsFC of the sensorimotor circuitry with an attention hub in explaining local hyperalgesia observed in patients with chronic neck pain.

Previous research has reported associations between M1 reorganization and impaired motor control in patients with chronic musculoskeletal pain (Chang et al., 2018) and differences in M1 representations of neck flexor muscles in patients with CINP compared to pain-free controls (Elgueta-Cancino et al., 2019). The precentral gyrus investigated in our study is the average of this large cortical region and hence it is unclear whether hyperalgesia is correlated with the entire precentral gyrus or specifically with the representation of the neck region in the motor homunculus. This is an interesting avenue for further research. Additionally, in previous research, enhanced rsFC of the right precentral gyrus -but then with the right posterior intraparietal sulcus- was also associated with pressure hyperalgesia in individuals with fibromyalgia (van Ettinger-Veenstra et al., 2020). Interestingly, our previous vertex-wise structural brain analyses revealed increased grey matter volume in the left superior parietal gyrus of patients with CINP compared to healthy controls, and cortical thickening of the left superior parietal gyrus in CINP compared to healthy controls and CWAD (De Pauw et al., 2019b). In addition, patients with CWAD showed smaller grey matter volume in the right precentral gyrus compared to healthy controls, and decreased grey matter volume in the precentral gyrus was observed in relation with worse performance on neuromuscular control (De Pauw et al., 2019b). Here, we extend these findings showing corresponding functional alterations in connectivity of this region with the bilateral M1 in relation to increased sensitivity to pressure pain in this population. Interestingly, these associations were present across the entire patient group, indicating they are not specific to a diagnosis. Such associations may even extend to other types of (chronic) pain, although this remains to be tested.

### 4.4 Considerations, clinical implications and recommendations for further research

In this study, a ROI-to-ROI-based rsFC approach was performed focusing on a set of predefined seeds derived from previous research in patients with chronic neck pain, in order to reduce the multiple comparisons problem (e.g., compared to a ROI-to-voxel approach). Thus, although our approach encompassed all regions that were relevant based on previous findings in patients with chronic neck pain, yet it did not include all possible combinations of brain regions, and hence was a mixture of explorative and driven by previous literature. The present results therewith add evidence to our previously published findings in this study sample of brain network changes during rest (De Pauw et al., 2019a), changes in grey matter morphology in patients with CWAD (Coppieters et al., 2017b, De Pauw et al., 2019b), and CINP (De Pauw et al., 2019), and changes in white matter structure only in patients with CWAD (Coppieters et al., 2018) compared to healthy pain-free persons. We can, however, not exclude the possibility that other circuitry is altered and/or is associated with sensory disturbances as well. Whole-brain approaches could shed more light on that. Lastly, note that as our data were acquired during rest, we cannot draw any direct conclusions on underlying mechanisms. To get direct insights in underlying neural mechanisms further studies could focus on acquiring functional data while performing QST in the MRI scanner in patients with chronic neck pain.

Our results emphasize the role of functional brain alterations in rest and their association with decreased endogenous pain inhibition, and associations between enhanced rsFC in sensorimotor circuitry and local hyperalgesia in women with CWAD and CINP. Accordingly, it can be recommended that therapy approaches for individuals with CWAD and CINP should take into account neuroplasticity of the central nervous system (being most pronounced in CWAD) and be mindful of the involvement of cognitive/affective neural circuitry in relation to pain sensitivity. Multi-modal treatment approaches, combining cognitive behavioral and physical therapy (e.g., pain neuroscience education plus cognition-targeted exercise therapy (Malfliet et al., 2017) would be especially relevant. Research already showed that connectivity changes in brain regions that are also identified in the current study, such as the amygdala with frontal cortex, seem to be characteristic of positive responses to interventions for chronic pain (Cunningham et al., 2019, Nahman-Averbuch et al., 2020, Simons et al., 2014). Future studies investigating the responsiveness of amygdala rsFC to changes following multi-modal treatment and associations with clinical improvements are essential in CINP and CWAD. Prospective longitudinal studies are warranted to gain insight in the role of these brain alterations in the transition from acute to chronic neck pain. And, further research examining neuroimaging-based pain biomarkers (Mackey et al., 2019) as an adjunct to self-reported, behavioral, and QST measures, also has the potential to advance personalized pain management in people with chronic neck pain.

### 4.5 Conclusion

Taken together, our findings demonstrate enhanced rsFC around a cluster comprising amygdala and pallidum as subcortical limbic and basal ganglia regions, and ventral frontal regions, including frontal operculum and orbitofrontal cortex. The most robust group difference was enhanced amygdala functional coupling during rest with the frontal operculum in women with CINP and CWAD compared to healthy controls, which was also associated with decreased endogenous pain inhibition across patients, and more CS-related symptoms within CINP patients. This enhanced fronto-limbic connectivity - typically involved in emotion (or threat) processing and regulation - and its association with diminished endogenous pain inhibition emphasizes the link between cognitive-affective and sensory modulations of pain in patients with chronic neck pain. In addition, independent of group differences, increased rsFC of the superior parietal cortex with bilateral precentral gyrus showed associations with more local hyperalgesia, pointing towards an important role for this sensorimotor circuitry in pain hypersensitivity in patients with chronic neck pain – which may even extend beyond neck pain.

## CRediT authorship contribution statement

**Iris Coppieters:** Conceptualization, Methodology, Software, Formal analysis, Investigation, Data curation, Writing - original draft, Writing - review & editing, Visualization, Funding acquisition. **Barbara Cagnie:** Conceptualization, Writing - review & editing, Supervision, Project administration, Funding acquisition. **Robby De Pauw:** Conceptualization, Investigation, Data curation, Writing - review & editing. **Mira Meeus:** Conceptualization, Writing - review & editing, Supervision, Project administration, Funding acquisition. **Inge Timmers:** Conceptualization, Methodology, Software, Formal analysis, Writing - review & editing, Visualization, Supervision.

## Declaration of Competing Interest

The authors declare that they have no known competing financial interests or personal relationships that could have appeared to influence the work reported in this paper.

## Footnotes

1 Note that all healthy controls included in the study reported a 0/10 verbal numeric rating scale for current pain intensity on the assessment day (see Fig. 1).
