# Supplementary material for: Enhanced amygdala-frontal operculum functional connectivity during rest in women with chronic neck pain: Associations with impaired conditioned pain modulation
Source: Neuroimage Clin. 2021 Mar 22;30:102638. doi: 10.1016/j.nicl.2021.102638 (PMC8053790; doi:10.1016/j.nicl.2021.102638)
Supplement: Supplementary data 1 [file mmc1.docx]

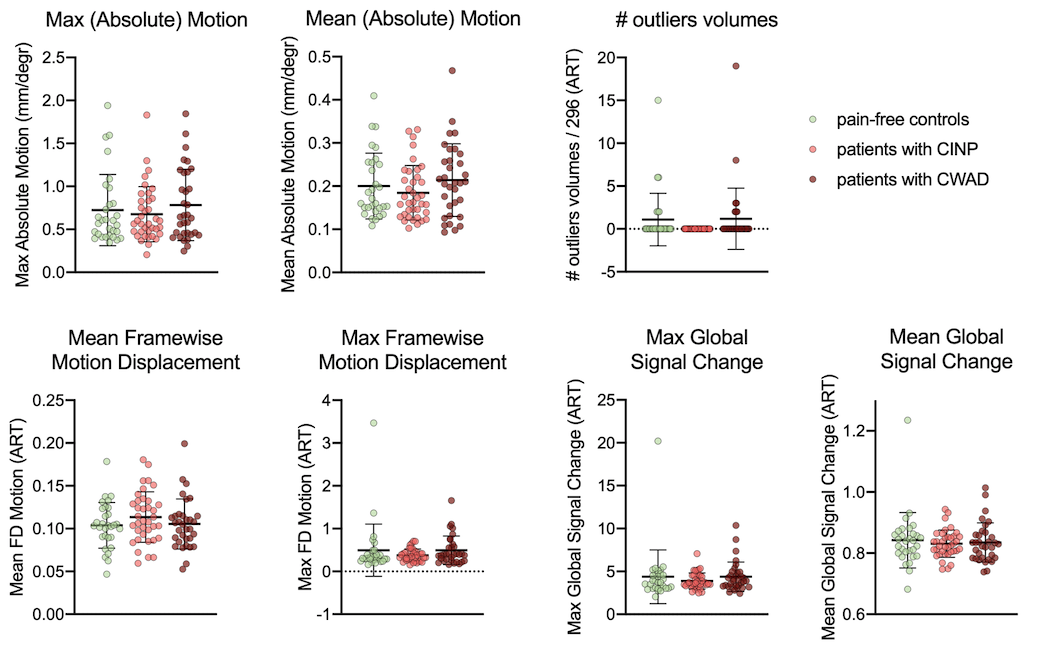


**Figure S1**. Overview of motion parameters in pain-free controls, patients with chronic idiopathic neck pain (CINP) and patients with chronic whiplash associated disorders (CWAD). Mean and standard deviation and values of individual participants are presented. No significant differences between groups were observed.

**Table S1.** Associations between rsFC and CSI and QST, and interaction effects with group.

|  | **Bivariate correlations** | | | **Linear Regression model** | |
| --- | --- | --- | --- | --- | --- |
|  | **All patients** | **CINP only** | **CWAD only** | **R^2^ change**^1^ | **Interaction term** |
| **Associations with group differences in rsFC** | | | | | |
| **left amygdala – left frontal operculum rsFC with CSI** | *r* = .30* | *r* = .47** | *r* = .03 | 5.4% | *β* = -1.09 (*t* = -1.99, *p* = .05) |
| **left amygdala – left frontal operculum rsFC with CPM quadriceps** | *r* = .31* | *r* = .29 | *r* = .20 | .2% | *β* = -.07 (*t* = -.36, *p* = .72) |
| **Associations between rsFC across all selected regions and QST measures** | | | | | |
| **left superior parietal cortex - left precentral gyrus rsFC with PPT trapezius** | *r* = -.39** | *r* = -.37* | *r* = -.51** | .6% | *β* = -.16 (*t* = -.68, *p* = .50) |
| **left superior parietal cortex - right precentral gyrus rsFC with PPT trapezius** | *r* = -.35** | *r* = -.23 | *r* = -.49** | 2.6% | *β* = -.34 (*t* = -1.40, *p* = .17) |

* *p* < .05, *** p* < .01, ^1^ R^2^ change between model 1 (QST measure and patient group as predictors) and model 2 (with added interaction term as product of patient group and QST measure). rsFC: resting-state functional connectivity, CPM: conditioned pain modulation, QST: quantitative sensory testing, PPT: pressure pain thresholds, CSI: Central Sensitization Inventory, CWAD: chronic whiplash associated disorders, CINP: chronic idiopathic neck pain.

**
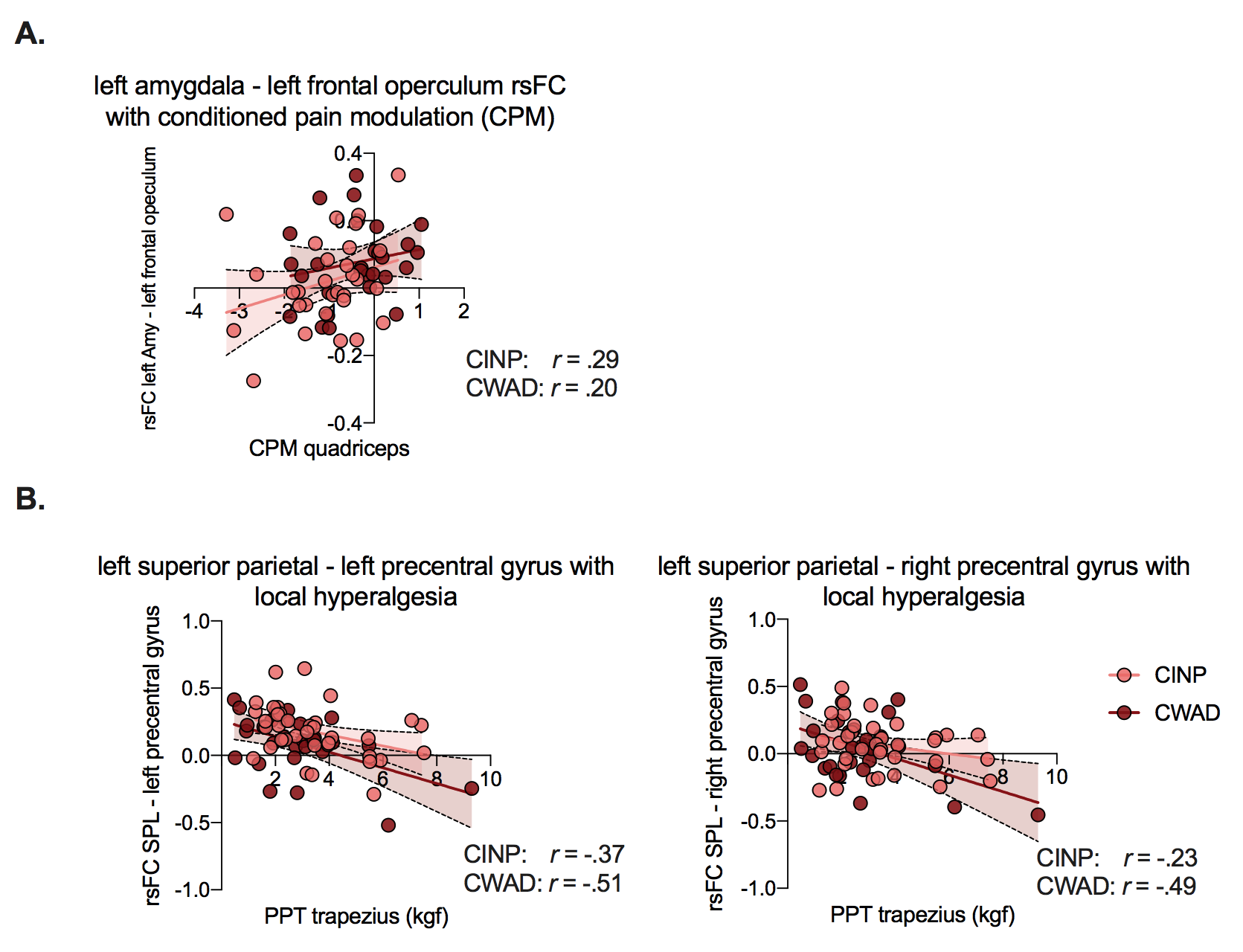
**

**Figure S2**. Scatterplot of associations between rsFC showing group differences and CPM taking CINP and CWAD separately, showing no significant interaction effect with group (A). Scatterplots of associations between higher local hyperalgesia (i.e., decreased PPTs), and increased rsFC between left superior parietal cortex and right and left precentral gyrus at the level of all selected regions taking CINP and CWAD separately, showing no significant interaction effect with group (B). PPT: pressure pain threshold, rsFC: resting-state functional connectivity, CPM: conditioned pain modulation, CWAD: chronic whiplash associated disorders, CINP: chronic idiopathic neck pain.
